# The Correlation between Chitin and Acidic Mammalian Chitinase in Animal Models of Allergic Asthma

**DOI:** 10.3390/ijms161126033

**Published:** 2015-11-16

**Authors:** Chia-Rui Shen, Horng-Heng Juang, Hui-Shan Chen, Ching-Jen Yang, Chia-Jen Wu, Meng-Hua Lee, Yih-Shiou Hwang, Ming-Ling Kuo, Ya-Shan Chen, Jeen-Kuan Chen, Chao-Lin Liu

**Affiliations:** 1 Department of Medical Biotechnology and Laboratory Science, College of Medicine, Chang Gung University, 259 Wen-Hwa 1st Road, Kweishan, Taoyuan 33302, Taiwan; crshen@mail.cgu.edu.tw (C.-R.S.); m9805024@stmail.cgu.edu.tw (H-S.C.); cjyang@mail.dcb.org.tw (C.-J.Y.); b9309033@stmail.cgu.edu.tw (M.-H.L.); d9601404@stmail.cgu.edu.tw (Y.-S.C.); 2 Graduate Institute of Biomedical Science, College of Medicine, Chang Gung University, 259 Wen-Hwa 1st Road, Kweishan, Taoyuan 33302, Taiwan; hhj143@mail.cgu.edu.tw (H.-H.J.); popycute@seed.net.tw (C.-J.W.); mingling@mail.cgu.edu.tw (M.-L.K.); 3 Chang Gung Immunology Consortium, Chang Gung Memorial Hospital and Chang Gung University, 5 Fu-Hsing Street, Kweishan, Taoyuan 33375, Taiwan; 4 Department of Anatomy, College of Medicine, Chang Gung University, 259 Wen-Hwa 1st Road, Kweishan, Taoyuan 33302, Taiwan; 5 Department of Chemical Engineering, Ming Chi University of Technology, 84 Gung-Juan Road, Taishan, New Taipei 24301, Taiwan; 6 Department of Microbiology, College of Medicine, Chang Gung University, 259 Wen-Hwa 1st Road, Kweishan, Taoyuan 33302, Taiwan; 7 Department of Medicine, College of Medicine, Chang Gung University, 259 Wen-Hwa 1st Road, Kweishan, Taoyuan 33302, Taiwan; ejubibi@cgmh.org.tw; 8 Department of Ophthalmology, Chang Gung Memorial Hospital, 5 Fu-Hsing Street, Kweishan, Taoyuan 33375, Taiwan; 9 Department of Environment and Biotechnology, Refining and Manufacturing Research Institute, CPC Corporation, Chiayi, 217 Min-Sheng S. Rd, Chiayi 60051, Taiwan; 078450@cpc.com.tw

**Keywords:** allergy, asthma, chitinase, promoter

## Abstract

Asthma is the result of chronic inflammation of the airways which subsequently results in airway hyper-responsiveness and airflow obstruction. It has been shown that an elicited expression of acidic mammalian chitinase (AMCase) may be involved in the pathogenesis of asthma. Our recent study has demonstrated that the specific suppression of elevated AMCase leads to reduced eosinophilia and Th2-mediated immune responses in an ovalbumin (OVA)-sensitized mouse model of allergic asthma. In the current study, we show that the elicited expression of AMCase in the lung tissues of both ovalbumin- and Der P2-induced allergic asthma mouse models. The effects of allergic mediated molecules on AMCase expression were evaluated by utilizing promoter assay in the lung cells. In fact, the exposure of chitin, a polymerized sugar and the fundamental component of the major allergen mite and several of the inflammatory mediators, showed significant enhancement on AMCase expression. Such obtained results contribute to the basis of developing a promising therapeutic strategy for asthma by silencing AMCase expression.

## 1. Introduction

The prevalence and severity of atopy diseases such as asthma have increased dramatically. In fact, the World Health Organization (WHO) has recognized asthma as a major public health concern. Asthma is characterized by chronic airway inflammation, along with the pathological helper T cell type 2 (Th2)-mediated immune responses. The production of allergen-specific IgE usually results in immediate-type hypersensitivity and is subsequently followed by late-phase responses including eosinophil recruitment, airway hyper-responsiveness (AHR), and mucus production. Eosinophilic inflammation driven by Th2-associated cytokines likely plays an important role in the development of asthma [1].

Chitin, a polymerized sugar and the fundamental component of the exoskeletons of invertebrates, is emerging as a potentially crucial and previously underappreciated environmental factor in asthma [2,3]. Specifically, the major allergens are mites and cockroaches, and chitin is their abundant component. In fact, a recent report detected chitin in home dust samples, and chitin concentrations were correlated with dust mite antigen, indicating a potential relationship between chitin exposure and asthma development/severity [4]. Chitinases comprise a group of related enzymes that are remarkably well preserved across mammalian species including humans [5,6,7]. Increasing evidence indicates that the heightened expression of chitinase (such as acidic mammalian chitinase (AMCase) plays a role in the development of asthma [8,9,10,11,12]. AMCase is hyper-expressed in the lung tissue of patients with asthma and in asthmatic animal models [5,8,9,13,14]. The induced expression of AMCase appeared to be found in lung epithelium and alveolar macrophages in ovalbumin-sensitized (OVA-sensitized) mice [8,9]. In addition, AMCase might be associated with Th2-mediated inflammatory responses [9,15,16,17,18] due to the significant hyper-expression of AMCase in IL-13-transgenic mice. In contrast, no AMCase expression was present in the IL-13-null mice, even with exposure to an allergen challenge [9], indicating an IL-13-dependent pathway. Although the inhibition of AMCase activity with inhibitors reduces the inflammation in the bronchoalveolar lavage fluid (BALF) and lung tissue, it appears that the reduction in AMCase activity failed to lead to consequent changes in IL-4 and/or IL-13 expression. Our laboratory adapted shRNA to knock down the expression of AMCase, and as such has demonstrated the significant suppression of allergic reactions [15].

The current study attempted to correlate exposure to chitin with allergic mediators and the expression of AMCase. We demonstrate the elicited expression of AMCase in lung tissue in an OVA-induced allergic asthma mouse model and in a Der P2-induced allergic asthma mice. In addition, the mouse AMCase promoter was cloned and constructed as a reporter vector. The effects of chitin, its derivative, and allergic mediated molecules were evaluated based on AMCase expression in lung cells.

## 2. Results and Discussion

### 2.1. Levels of AMCase in the Lungs of Ovalbumin (OVA)- or Dermatophagoides Pteronyssinus Group II Allergen (Der P2)-Sensitized Mice

**Figure 1:**
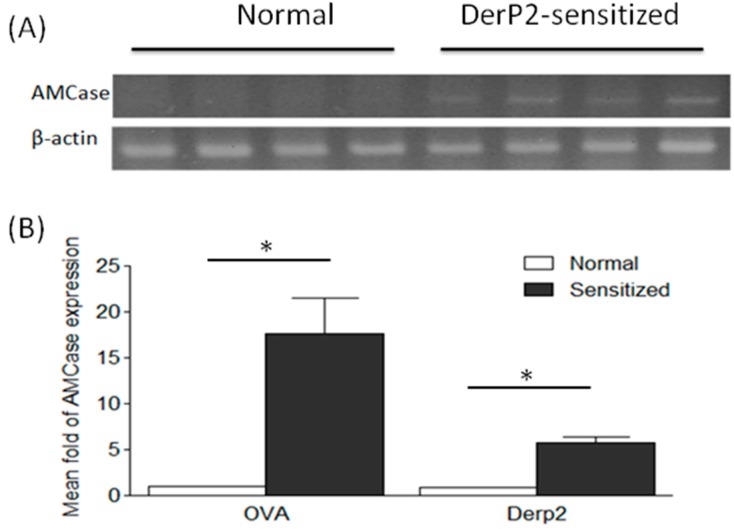
The elicited AMCase expression levels in the mice sensitized with OVA and Der-P2. The lung samples were harvested from normal saline controls, ovalbumin (OVA)- or Dermatophagoides pteronyssinus group II allergen (Der P2) mice at day 28. (The number of mice in each group was more than four.) Their AMCase expression was quantified via (**A**) RT-PCR and (**B**) real-time PCR (* *p* < 0.05, normal *vs.* sensitized, nonparametric Mann-Whitney *U* test).

To determine whether the elevated levels of AMCase expression specifically occurred in the airways of OVA- or Der P2-sensitized mice, we harvested and analyzed lung tissues 24 h after the last allergen challenge. Figure 1A shows that the elicited levels of AMCase expression were found in the lungs of Der P2-sensitized mice and not in those of normal controls. Since semi-quantitative data were considered to be non-parametric, the Mann–Whitney *U* test was utilized to compare the differences of AMCase expression in the lung samples of OVA- and Der P2-sensitized mice. Notably, 17- and five-fold increases in the AMCase mRNA levels were found in the lung tissues of OVA- and Der P2-sensitized mice, respectively (Figure 1B). In addition, no major differences were found with regard to AMCase expression in the peritoneal cells from sensitized animals compared with control mice treated with normal saline (data not shown). Similar findings were obtained when examining the AMCase protein levels using Western blot analysis (data not shown). Hence, the hyperexpression of AMCase was confined chiefly to the airways.

### 2.2. AMCase Reporter Vector Construction and AMCase Expression

**Figure 2:**
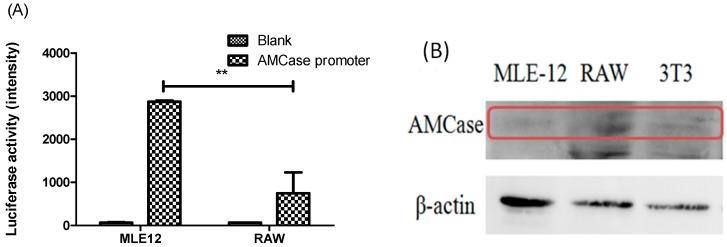
The expression of AMCase. (**A**) Reporter vector containing the AMCase promoter and encoding luciferase was transfected to MLE (mouse lung epithelial)-12 and RAW macrophage cells, and luciferase activity was measured after 48 h of transfection (** *p* < 0.01, MLE (+AMCase promoter) *vs.* RAW (+AMCase promoter), unpaired *t*-test); (**B**) The protein levels of AMCase (shown in the red rectangle) in MLE-12, RAW264.7, and BALB/c 3T3 fibroblast cells were analyzed using Western blotting.

The specific mouse AMCase promoter was cloned using PCR accordingly, and the reporter vector pGL3 was constructed containing the AMCase promoter and encoding luciferase. Therefore, AMCase expression in the cells can be evaluated by measuring the luciferase activity in the cells transiently transfected with the reporter vector. Figure 2A shows significantly higher levels of AMCase expression in mouse lung epithelium MLE-12 cells than the RAW macrophages. Such expression profiles were confirmed using Western blot analysis with anti-AMCase antibody for specific detection (Figure 2B). In fact, detected levels of AMCase expression in most cell lines were low using either Western blot or real-time PCR assays. To confirm the results demonstrated by the reporter luciferase assays, we used nested RT-PCR and obtained similar findings (data not shown).

### 2.3. The Effects of Chitin on AMCase Expression

**Figure 3:**
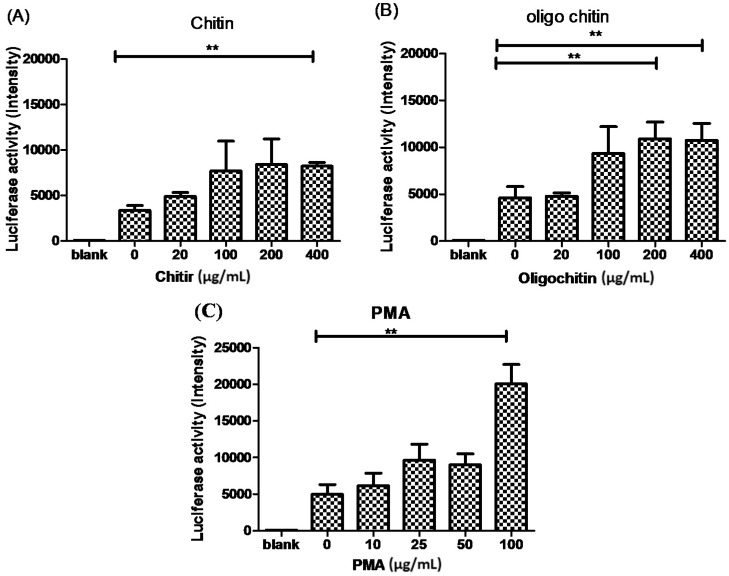
The effect of chitin derivative on the expression of AMCase in MLE-12 cells. The plasmid construct containing AMCase promoter and encoding luciferase was transfected to MLE-12 and RAW cells, and luciferase activity was measured after 48 h of incubation. At 24 h post-transfection, different doses of (**A**) chitin; (**B**) oligo-chitin; and (**C**) PMA were added to the cultures (** *p* < 0.01, paired *t*-test).

To correlate the exposure of chitin with the expression of AMCase and to evaluate whether such a correlation contributes to the development of allergic asthma, we identified the effects of chitin, its derivative, and allergic mediated molecules on AMCase expression in lung cells. The results are summarized in Figure 3. The levels of AMCase expression are presented by luciferase activity and shown in relative light units. Figure 3A shows that a relatively low level of AMCase was expressed in MLE-12 cells, whereas the addition of chitin induced AMCase expression. As doses of the chitin challenge were raised, the levels of AMCase increased. Similar patterns were found with the addition of a chitin derivative (oligo-chitin; Figure 3B). This study also used phorbol 12-myristate 13-acetate (PMA), as a positive control to stimulate AMCase expression (Figure 3C).

This study demonstrated that AMCase was increased in the lung tissues of mice primed and sensitized with OVA or Der-P2. In particular, AMCase was highly expressed in OVA-sensitized mice, and that might be due to the severe asthmatic symptoms found in the OVA-induced asthmatic mouse model [8,19]. In fact, our previous and current work and also the work of Zhu *et al.* in 2004 [9], Bierbaum *et al.* in 2005 [13], and Chupps *et al.* in 2007 [14] demonstrated the increased chitinase activity in the asthmatic mouse and clinical human samples. In particular, Zhu *et al.* found that the expression of AMCase was induced both in murine lung epithelium and in alveolar macrophages [9]. Moreover, Chupps *et al.* indicated that in patients with severe asthma, the chitinase-like protein was not only found in the lungs but also in the circulation [14]. Therefore, in this study, we tested the AMCase activity in several different cell types. We found that only the tested MLE-12 cells, but not the 3T3 or RAW cells, appeared to harbor the chitinase activity via the promoter assay. However, it still cannot be excluded that proteins with chitinase activity other than AMCase might exist in those cells.

Additionally, the Western blot analysis showed that AMCase was expressed at a fairly low level in MLE-12 cells. However, using the more sensitive promoter assay, we showed that AMCase was expressed at a relatively high level in MLE-12 cells but not in RAW cells. In fact, in our earlier study [8], we had demonstrated an enzyme functional assay result for AMCase to confirm the elicited chitinase function in OVA-sensitized asthmatic animals. Finally, we have revealed that chitin and its derivative oligo-chitin were able to stimulate the expression of AMCase. The results coincide with plant and microorganism chitinase promotors induced by chitin and its derivative [19,20,21]. Although studies of evaluating allergic mediators remain ongoing, these results provide some evidence basis of AMCase that might contribute to the current knowledge regarding the participants for allergic disease.

## 3. Materials and Methods

### 3.1. Cell Culture

The cell lines mouse lung epithelium (MLE)-12, BALB/c 3T3, and RAW 264.7 cells were cultured and maintained in Dulbecco’s modified Eagle’s medium (DMEM), supplemented with 5% fetal bovine serum (FBS) at 37 °C, along with 5% CO_2_ in humidified air.

### 3.2. Asthmatic Animal Induction Protocol

The animal protocols were approved by the Institutional Animal Care and Use Committee (IACUC) of Chang Gung University (Taoyuan, Taiwan), as published elsewhere. The recombinant chicken OVA was purchased from Sigma (St. Louis, MO, USA), and the Dermatophagoides pteronyssinus group II allergen (Der P2) was obtained from a yeast clone, as previously described [19]. Specific pathogen-free female BALB/cByJNarl mice were purchased from the National Laboratory Animal Center (Taipei, Taiwan). Mice aged 6–8 weeks were used in the study. The mice were injected intraperitoneally with recombinant chicken OVA (20 μg) or Der P2 (40 μg) mixed with aluminum hydroxide on experimental days 1, 7, and 14. Thereafter, the mice were challenged with the same allergens on day 20 and 27, and then sacrificed on day 28. The lung tissue was removed and harvested for further use. The animals receiving normal saline only served as the control group.

### 3.3. Real-Time Polymerase Chain Reaction (PCR)

The expressions of AMCase and humanized Renilla green fluorescent protein (hrGFP) were analyzed using real-time PCR with a LightCycler PCR system (Roche, Indianapolis, IN). AMCase was amplified using the following primers: forward, 5′-TGGACACACCTTCATCCTGA-3′; and reverse, 5′-AACA AGCCCTGCTTGACAAT-3′. After initial denaturation at 95 °C for 10 min, 45 cycles were performed at 95 °C for 10 s, 50 °C for 10 s, and 72 °C for 10 s. The reaction parameters for hrGFP amplification (forward primer, 5′-ATGGTGAGCAAGCAGATCCTG-3′; reverse, 5′-GGTGCGCTCGTACACGAAGCC-3′) were as follows: initial denaturation at 95 °C for 10 min, followed by 35 cycles at 95 °C for 10 s, 50 °C for 10 s, and 72 °C for 10 s. Actin as a housekeeping gene used the same protocol as hrGFP, but the primers were as followed: forward primer, 5′-GAAACTACATTCAAT TCCATC-3′; reverse primer, 5′-CTAGAAGCACTTGCGGT GCAC-3′.

### 3.4. Western Blot Analysis

The tissue and cell samples harvested for the AMCase analysis were prepared with lysis buffer for Western blot analysis. After separation via sodium dodecyl sulfate-polyacrylamide gel electrophoresis (SDS-PAGE), the samples were identified using incubation with rabbit antimouse AMCase antibodies (Santa Cruz Biotech, Dallas, TX, USA) and developed with an enhanced chemiluminescence (ECL) detection kit (GE Healthcare Life Sciences, Piscataway, NJ, USA). A monoclonal antibody against β-actin was used as a control.

### 3.5. Mouse AMCase Reporter Vector Construction and Transient Gene Expression Assay

A 7.6-kb gene fragment of the mAMCase was excised from the Bacterial Artificial Chromosome (BAC) clone RP23-62K10 (Invitrogen, Carlsbad, CA, USA) and ligated into the pGEM-5 vector (Promega BioScience, Madison, WI, USA). The fragment containing the promoter and the 5′-flanking region of the mAMCase gene (-6667 to -1) was synthesized by PCR amplification using T7 and mAMCasepromR (5′-CTCGAGTTCCTCCCGTACTGCTCCAGG-3′) oligonucleotide primers, and ligated into the pGL3-Basic vector (Promega BioScience).

Cells were transiently transfected using TurboFect transfection reagent (Thermo Scientific, Waltham, MA, USA.) as described elsewhere [20]. Briefly, the cells at 1 × 10^5^ cells/well in six-well plates were transfected with 3 μL/well of TurboFect plus 3 μg/well of reporter vector. After culturing for 24 h, the cells, which were transfected with the luciferase reporter vector, were treated with different doses of chitin, its derivative, and allergic mediators in DMEM with 5% FBS for an additional 24 h. The cell lysate (100 μL) was applied for the luciferase assay, and the luciferase activity was determined in relative light units using a LumiCount Luminometer (Packard BioScience, Meriden, CT, USA). Additionally, whenever appropriate, the normalization control plasmid pCMVSPORTβgal was utilized, and then the luciferase activity could be adjusted for efficient transfection. Oligo chitin was obtained from Lytone Biotech Co., Taipei, Taiwan.

### 3.6. Statistical Analyses

The results are presented as mean ± SEM. Statistical analysis was performed by GraphPad Prism software (version 5.0, San Diego, CA, USA). The nonparametric Mann-Whitney *U* test, and the paired or unpaired *t*-test was used to evaluate *p*-values. *p*-values < 0.05 were defined as statistically significant.

## 4. Conclusions

The expression of AMCase was demonstrated to be elicited in the lung tissues of both OVA- and Der P2-induced allergic asthma mouse models. Also, chitin, its derivative, and the inflammatory mediators appeared to affect the AMCase expression in the lung cells. These findings suggest a crucial role of AMCase in allergic asthma. However, a large-scale study as well as more practical approaches are warranted for analyzing clinical samples or deriving benefits for clinical medicine.
